# Supplementary material for: HIVprotI: an integrated web based platform for prediction and design of HIV proteins inhibitors
Source: J Cheminform. 2018 Mar 9;10:12. doi: 10.1186/s13321-018-0266-y (PMC5845081; doi:10.1186/s13321-018-0266-y)
Supplement: Supplementary file 2 — Additional file 2. Source code of HIVProtI web server. [file 13321_2018_266_MOESM2_ESM.zip › HIVprotI_Source-code/hivprotI/weka-3-6-6/doc/weka/classifiers/meta/MultiClassClassifier.html]

MultiClassClassifier


---


|  |  |  |
| --- | --- | --- |
| **Overview** \| **Package** \| **Class** \| **Tree** \| **Deprecated** \| **Index** \| **Help** \| **Weka's home** | |  |
| **PREV CLASS**   **NEXT CLASS** | **FRAMES**    **NO FRAMES**     **All Classes** |  |
| SUMMARY: NESTED \| FIELD \| CONSTR \| METHOD | DETAIL: FIELD \| CONSTR \| METHOD |  |


---


## weka.classifiers.meta Class MultiClassClassifier

```
java.lang.Object
  weka.classifiers.Classifier
      weka.classifiers.SingleClassifierEnhancer
          weka.classifiers.RandomizableSingleClassifierEnhancer
              weka.classifiers.meta.MultiClassClassifier
```

**All Implemented Interfaces:**: java.io.Serializable, java.lang.Cloneable, CapabilitiesHandler, OptionHandler, Randomizable, RevisionHandler

---

``` public class MultiClassClassifier extends RandomizableSingleClassifierEnhancer implements OptionHandler ```

A metaclassifier for handling multi-class datasets with 2-class classifiers. This classifier is also capable of applying error correcting output codes for increased accuracy.


Valid options are:

```
 -M <num>
  Sets the method to use. Valid values are 0 (1-against-all),
  1 (random codes), 2 (exhaustive code), and 3 (1-against-1). (default 0)
```

```
 -R <num>
  Sets the multiplier when using random codes. (default 2.0)
```

```
 -P
  Use pairwise coupling (only has an effect for 1-against1)
```

```
 -S <num>
  Random number seed.
  (default 1)
```

```
 -D
  If set, classifier is run in debug mode and
  may output additional info to the console
```

```
 -W
  Full name of base classifier.
  (default: weka.classifiers.functions.Logistic)
```

```
 Options specific to classifier weka.classifiers.functions.Logistic:
```

```
 -D
  Turn on debugging output.
```

```
 -R <ridge>
  Set the ridge in the log-likelihood.
```

```
 -M <number>
  Set the maximum number of iterations (default -1, until convergence).
```

**Version:**
:   $Revision: 1.48 $

**Author:**
:   Eibe Frank (eibe@cs.waikato.ac.nz), Len Trigg (len@reeltwo.com), Richard Kirkby (rkirkby@cs.waikato.ac.nz)

**See Also:**: Serialized Form

---

| **Field Summary** | |
| --- | --- |
| `static int` | `METHOD_1_AGAINST_1`             1-against-1 |
| `static int` | `METHOD_1_AGAINST_ALL`             1-against-all |
| `static int` | `METHOD_ERROR_EXHAUSTIVE`             exhaustive correction code |
| `static int` | `METHOD_ERROR_RANDOM`             random correction code |
| `static Tag[]` | `TAGS_METHOD`             The error correction modes |


**Constructor Summary**

`MultiClassClassifier()`
  
          Constructor.


| **Method Summary** | |
| --- | --- |
| `void` | `buildClassifier(Instances insts)`             Builds the classifiers. |
| `double[]` | `distributionForInstance(Instance inst)`             Returns the distribution for an instance. |
| `Capabilities` | `getCapabilities()`             Returns default capabilities of the classifier. |
| `SelectedTag` | `getMethod()`             Gets the method used. |
| `java.lang.String[]` | `getOptions()`             Gets the current settings of the Classifier. |
| `double` | `getRandomWidthFactor()`             Gets the multiplier when generating random codes. |
| `java.lang.String` | `getRevision()`             Returns the revision string. |
| `boolean` | `getUsePairwiseCoupling()`             Gets whether to use pairwise coupling with 1-vs-1 classification to improve probability estimates. |
| `java.lang.String` | `globalInfo()` |
| `double[]` | `individualPredictions(Instance inst)`             Returns the individual predictions of the base classifiers for an instance. |
| `java.util.Enumeration` | `listOptions()`             Returns an enumeration describing the available options |
| `static void` | `main(java.lang.String[] argv)`             Main method for testing this class. |
| `java.lang.String` | `methodTipText()` |
| `static double[]` | `pairwiseCoupling(double[][] n, double[][] r)`             Implements pairwise coupling. |
| `java.lang.String` | `randomWidthFactorTipText()` |
| `void` | `setMethod(SelectedTag newMethod)`             Sets the method used. |
| `void` | `setOptions(java.lang.String[] options)`             Parses a given list of options. |
| `void` | `setRandomWidthFactor(double newRandomWidthFactor)`             Sets the multiplier when generating random codes. |
| `void` | `setUsePairwiseCoupling(boolean p)`             Set whether to use pairwise coupling with 1-vs-1 classification to improve probability estimates. |
| `java.lang.String` | `toString()`             Prints the classifiers. |
| `java.lang.String` | `usePairwiseCouplingTipText()` |

**Methods inherited from class weka.classifiers.RandomizableSingleClassifierEnhancer**

`getSeed, seedTipText, setSeed`

**Methods inherited from class weka.classifiers.SingleClassifierEnhancer**

`classifierTipText, getClassifier, setClassifier`

**Methods inherited from class weka.classifiers.Classifier**

`classifyInstance, debugTipText, forName, getDebug, makeCopies, makeCopy, setDebug`

**Methods inherited from class java.lang.Object**

`equals, getClass, hashCode, notify, notifyAll, wait, wait, wait`

**Field Detail**

### METHOD\_1\_AGAINST\_ALL

```
public static final int METHOD_1_AGAINST_ALL
```

:   1-against-all

    **See Also:**: Constant Field Values

---


### METHOD\_ERROR\_RANDOM

```
public static final int METHOD_ERROR_RANDOM
```

:   random correction code

    **See Also:**: Constant Field Values

---


### METHOD\_ERROR\_EXHAUSTIVE

```
public static final int METHOD_ERROR_EXHAUSTIVE
```

:   exhaustive correction code

    **See Also:**: Constant Field Values

---


### METHOD\_1\_AGAINST\_1

```
public static final int METHOD_1_AGAINST_1
```

:   1-against-1

    **See Also:**: Constant Field Values

---


### TAGS\_METHOD

```
public static final Tag[] TAGS_METHOD
```

:   The error correction modes


**Constructor Detail**

### MultiClassClassifier

```
public MultiClassClassifier()
```

:   Constructor.


**Method Detail**

### getCapabilities

```
public Capabilities getCapabilities()
```

:   Returns default capabilities of the classifier.

    :   **Specified by:**: `getCapabilities` in interface `CapabilitiesHandler` **Overrides:**: `getCapabilities` in class `SingleClassifierEnhancer`
    :   **Returns:**: the capabilities of this classifier **See Also:**: `Capabilities`

---


### buildClassifier

```
public void buildClassifier(Instances insts)
                     throws java.lang.Exception
```

:   Builds the classifiers.

    :   **Specified by:**: `buildClassifier` in class `Classifier`
    :   **Parameters:**: `insts` - the training data. **Throws:**: `java.lang.Exception` - if a classifier can't be built

---


### individualPredictions

```
public double[] individualPredictions(Instance inst)
                               throws java.lang.Exception
```

:   Returns the individual predictions of the base classifiers
    for an instance. Used by StackedMultiClassClassifier.
    Returns the probability for the second "class" predicted
    by each base classifier.

    :   **Parameters:**: `inst` - the instance to get the prediction for **Returns:**: the individual predictions **Throws:**: `java.lang.Exception` - if the predictions can't be computed successfully

---


### distributionForInstance

```
public double[] distributionForInstance(Instance inst)
                                 throws java.lang.Exception
```

:   Returns the distribution for an instance.

    :   **Overrides:**: `distributionForInstance` in class `Classifier`
    :   **Parameters:**: `inst` - the instance to get the distribution for **Returns:**: the distribution **Throws:**: `java.lang.Exception` - if the distribution can't be computed successfully

---


### toString

```
public java.lang.String toString()
```

:   Prints the classifiers.

    :   **Overrides:**: `toString` in class `java.lang.Object`
    :   **Returns:**: a string representation of the classifier

---


### listOptions

```
public java.util.Enumeration listOptions()
```

:   Returns an enumeration describing the available options

    :   **Specified by:**: `listOptions` in interface `OptionHandler` **Overrides:**: `listOptions` in class `RandomizableSingleClassifierEnhancer`
    :   **Returns:**: an enumeration of all the available options

---


### setOptions

```
public void setOptions(java.lang.String[] options)
                throws java.lang.Exception
```

:   Parses a given list of options.


    Valid options are:

    ```
     -M <num>
      Sets the method to use. Valid values are 0 (1-against-all),
      1 (random codes), 2 (exhaustive code), and 3 (1-against-1). (default 0)
    ```

    ```
     -R <num>
      Sets the multiplier when using random codes. (default 2.0)
    ```

    ```
     -P
      Use pairwise coupling (only has an effect for 1-against1)
    ```

    ```
     -S <num>
      Random number seed.
      (default 1)
    ```

    ```
     -D
      If set, classifier is run in debug mode and
      may output additional info to the console
    ```

    ```
     -W
      Full name of base classifier.
      (default: weka.classifiers.functions.Logistic)
    ```

    ```
     Options specific to classifier weka.classifiers.functions.Logistic:
    ```

    ```
     -D
      Turn on debugging output.
    ```

    ```
     -R <ridge>
      Set the ridge in the log-likelihood.
    ```

    ```
     -M <number>
      Set the maximum number of iterations (default -1, until convergence).
    ```

    :   **Specified by:**: `setOptions` in interface `OptionHandler` **Overrides:**: `setOptions` in class `RandomizableSingleClassifierEnhancer`
    :   **Parameters:**: `options` - the list of options as an array of strings **Throws:**: `java.lang.Exception` - if an option is not supported

---


### getOptions

```
public java.lang.String[] getOptions()
```

:   Gets the current settings of the Classifier.

    :   **Specified by:**: `getOptions` in interface `OptionHandler` **Overrides:**: `getOptions` in class `RandomizableSingleClassifierEnhancer`
    :   **Returns:**: an array of strings suitable for passing to setOptions

---


### globalInfo

```
public java.lang.String globalInfo()
```

:   **Returns:**: a description of the classifier suitable for displaying in the explorer/experimenter gui

---


### randomWidthFactorTipText

```
public java.lang.String randomWidthFactorTipText()
```

:   **Returns:**: tip text for this property suitable for displaying in the explorer/experimenter gui

---


### getRandomWidthFactor

```
public double getRandomWidthFactor()
```

:   Gets the multiplier when generating random codes. Will generate
    numClasses \* m\_RandomWidthFactor codes.

    :   **Returns:**: the width multiplier

---


### setRandomWidthFactor

```
public void setRandomWidthFactor(double newRandomWidthFactor)
```

:   Sets the multiplier when generating random codes. Will generate
    numClasses \* m\_RandomWidthFactor codes.

    :   **Parameters:**: `newRandomWidthFactor` - the new width multiplier

---


### methodTipText

```
public java.lang.String methodTipText()
```

:   **Returns:**: tip text for this property suitable for displaying in the explorer/experimenter gui

---


### getMethod

```
public SelectedTag getMethod()
```

:   Gets the method used. Will be one of METHOD\_1\_AGAINST\_ALL,
    METHOD\_ERROR\_RANDOM, METHOD\_ERROR\_EXHAUSTIVE, or METHOD\_1\_AGAINST\_1.

    :   **Returns:**: the current method.

---


### setMethod

```
public void setMethod(SelectedTag newMethod)
```

:   Sets the method used. Will be one of METHOD\_1\_AGAINST\_ALL,
    METHOD\_ERROR\_RANDOM, METHOD\_ERROR\_EXHAUSTIVE, or METHOD\_1\_AGAINST\_1.

    :   **Parameters:**: `newMethod` - the new method.

---


### setUsePairwiseCoupling

```
public void setUsePairwiseCoupling(boolean p)
```

:   Set whether to use pairwise coupling with 1-vs-1
    classification to improve probability estimates.

    :   **Parameters:**: `p` - true if pairwise coupling is to be used

---


### getUsePairwiseCoupling

```
public boolean getUsePairwiseCoupling()
```

:   Gets whether to use pairwise coupling with 1-vs-1
    classification to improve probability estimates.

    :   **Returns:**: true if pairwise coupling is to be used

---


### usePairwiseCouplingTipText

```
public java.lang.String usePairwiseCouplingTipText()
```

:   **Returns:**: tip text for this property suitable for displaying in the explorer/experimenter gui

---


### pairwiseCoupling

```
public static double[] pairwiseCoupling(double[][] n,
                                        double[][] r)
```

:   Implements pairwise coupling.

    :   **Parameters:**: `n` - the sum of weights used to train each model: `r` - the probability estimate from each model **Returns:**: the coupled estimates

---


### getRevision

```
public java.lang.String getRevision()
```

:   Returns the revision string.

    :   **Specified by:**: `getRevision` in interface `RevisionHandler` **Overrides:**: `getRevision` in class `Classifier`
    :   **Returns:**: the revision

---


### main

```
public static void main(java.lang.String[] argv)
```

:   Main method for testing this class.

    :   **Parameters:**: `argv` - the options


---


|  |  |  |
| --- | --- | --- |
| **Overview** \| **Package** \| **Class** \| **Tree** \| **Deprecated** \| **Index** \| **Help** \| **Weka's home** | |  |
| **PREV CLASS**   **NEXT CLASS** | **FRAMES**    **NO FRAMES**     **All Classes** |  |
| SUMMARY: NESTED \| FIELD \| CONSTR \| METHOD | DETAIL: FIELD \| CONSTR \| METHOD |  |


---
